# Are autopsies on minors a taboo?: The experience of Milan in a 19-year retrospective study

**DOI:** 10.1007/s00414-023-03114-x

**Published:** 2023-11-07

**Authors:** Stefano Tambuzzi, Graziano Crudele, Lidia Maggioni, Federica Collini, Sara Tunesi, Adriano Decarli, Antonio Giampiero Russo, Cristina Cattaneo

**Affiliations:** 1 Department of Biomedical Sciences for Health, Institute of Forensic Medicine, University of Milan, Luigi Mangiagalli Street, 37, 20133 Milan, Italy; 2 Department of Health Sciences, University of Eastern Piedmont Amedeo Avogadro, Novara, Italy; 3 Epidemiology Unit, Agency for Health Protection of Milan, Milan, Italy

**Keywords:** Minors, Underage victims, Non-natural deaths, Forensic autopsy

## Abstract

Forensic autopsy is an important tool for the proper management of non-natural deaths in minors. However, it seems that autopsy in minors is a practice which may not be performed routinely. In this framework, we conducted a study analyzing autopsies of minors (under 18 years of age in Italy) performed at the Institute of Forensic Medicine in Milan in the period 2001–2019. For the period 2015–2019, we extrapolated all deaths due to non-natural causes in minors to investigate how many and which of these deaths were not subjected to forensic autopsy. Of the total, 344 minors (235 males and 109 females) underwent autopsies, with an overall downward trend of about 80% since 2004. Most autopsies occurred between the ages of 0 and 1 year, and the fewest between the ages of 5 and 9 years. The place of death was home in most cases, and accidental death was most common, followed by natural death, suicide, and homicide, with prevalence varying by age group. Blunt force trauma predominated among accidental death in all age groups, followed by asphyxia. Similar findings were observed for suicides, although there was a more differentiated pattern for suicides between the ages of 15 and 17 years. Among homicides, blunt force trauma, asphyxia, and gunshot wounds were fairly evenly distributed across all age groups. Between 2015 and 2019, a total of 86 minors died of a non-natural cause, and a forensic autopsy was performed in only 33 cases (38%). Our data shows that fewer and fewer autopsies are being performed over the last years, which indicates a dangerous lack of forensic investigation of children and adolescent deaths, with enormous implications for prevention of child abuse.

## Introduction

Children and adolescents are among the most vulnerable population groups and for this reason are at higher risk of both direct and indirect violence [1]. In the domestic sphere alone, approximately 275 million children experience some form of violence each year [2], and the number of children who are victims of neglect and/or non-accidental trauma is also often underestimated [3–6]. Regardless of protective or preventive orientation, systems often fail to protect minors, resulting in the worst possible outcomes, such as death. According to UNICEF research, in the United States alone, 1,750 children died as a result of abuse and neglect in 2020, and nearly 3,500 children die each year in developed countries from physical abuse and neglect [7]. The World Health Organization (WHO) estimated homicide rates per 100.000 population in 2017 among subjects aged 0 to 17 years in different regions, ranging from a low of 0.5% in the Western Pacific to a high of 5.8% in the Americas [8]. However, the greater frailty of children and adolescents also leads to an increased risk of accidental traumatic events and non-natural causes of death (accident or suicide). In particular, injury-related causes remain the leading cause of death in children and adolescents [9, 10]. Overall, there were nearly 1 million deaths in children and adolescents due to non-natural causes worldwide in 2019 alone [1, 9, 11]. These numbers are proportionally higher in developed countries, where deaths in children and adolescents from natural causes (infectious diseases and cancer) have become less common [9, 10]. In other words, the number of non-natural deaths (especially accidental deaths) is high also in less developed countries, but at the same time the number of natural deaths is very high as well [9, 11]. Despite these dramatically high numbers, the question of whether cases of children dying from violent traumatic causes are adequately investigated from a forensic perspective through judicial autopsies has been largely overlooked in the literature. While several papers generally address infants and young children, there are few forensic autopsy studies that address older children and adolescents; furthermore, the few papers on adolescent deaths address only specific aspects [12]. In particular, there is hardly any autopsy studies on the age range of 4 to 9 years [13]. However, the usefulness of autopsy of minors has been repeatedly confirmed: autopsy in this group of persons, in fact, allows the study of the actual occurrence of diseases or acts of violence for epidemiological purposes, but also and above all the search for signs of maltreatment and other medico-legal information [14]. In the specific case of Italy, data on child and adolescent maltreatment are not readily available [15], and mortality due to violence is also underestimated [16, 17]. In our experience, autopsies are not always ordered even in cases of non-natural deaths of children and adolescents. In light of this general lack of knowledge and awareness of this phenomenon, we conducted a study analyzing the number of autopsies of minors (under 18 years of age) at the Institute of Forensic Medicine in Milan in the period 2001–2019 and comparing it with the total number of deaths according to ISTAT (Italian National Statistical Institute) data. Subsequently, thanks to the collaboration with the Local Health Authority, called Agency for Health Protection of Milan (ATS—*Agenzia di Tutela della Salute*), the study of underage deaths due to non-natural causes in the period 2015–2019 was pursued more in depth. The aim was to investigate how many and which violent deaths were not subjected to forensic autopsy. More generally, non-natural deaths among minors, especially homicides and suicides, can reflect the social stability of a region or even a country and provide important information for the protection of socially disadvantaged groups.

## Material and methods

The Institute of Forensic Medicine in Milan is located in one of the most important cities in northern Italy and it is unique for the entire city and its surroundings. Therefore, all deaths in which a pathological or forensic autopsy is deemed necessary are referred to it. The only exceptions are certain diagnostic autopsies performed by clinical pathologists in hospitals, which do not include deaths of non-natural causes. For these reasons, and considering the population of Milan (about 1.3 million), the Institute of Forensic Medicine has a large influx of deceased, almost representative of the entire local population. From the autopsy archive, all cases of autopsies performed on minors (0–17 years, since the age of majority in Italy is reached at 18 years) in the selected period (2001–2019) were extrapolated, focusing on age, type of autopsy performed, methods of injury, manner and cause of death. The latter diagnoses were made by forensic pathologists after a systematic evaluation of death scene information, gross autopsy, and laboratory analyses, when appropriate.

To deepen the study, we asked the collaboration of ATS, since it has a privileged point of view being able to provide epidemiological-statistical information on the population residing in the municipality. Moreover, ATS also maintains the Register of Causes of Death (ReNCaM), which is based on the ISTAT [18] death records for reporting the cause of death in the entire population of Milan. It is mandatory that these forms be filled out by a physician in order to proceed with the burial of the deceased, as provided for in the Ordinance of the Mortuary Police (Presidential Decree 285 of September 10, 1990) [19]. In particular, we wanted to investigate how many minors actually died, also of non-natural causes, in the whole region of Milan (surroundings included) in the period from 2001 to 2019, in order to compare this information with the number of minors who underwent an autopsy. For all the analyses we decided to exclude 2020 as the year of the pandemic.

Finally, for a selected time period (2015–2019), we extracted anonymized information from ReNCaM records on age, method of injury, and manner of death for all minors who died of non-natural causes. Minors who died of non-natural causes but were not subjected to a forensic autopsy were identified by matching against the Institute of Forensic Medicine database.

All data collected were analyzed using descriptive statistics.

The study was approved by the Ethics Committee of the University of Milan (approval n. 26/2022).

## Results

**Fig. 1:**
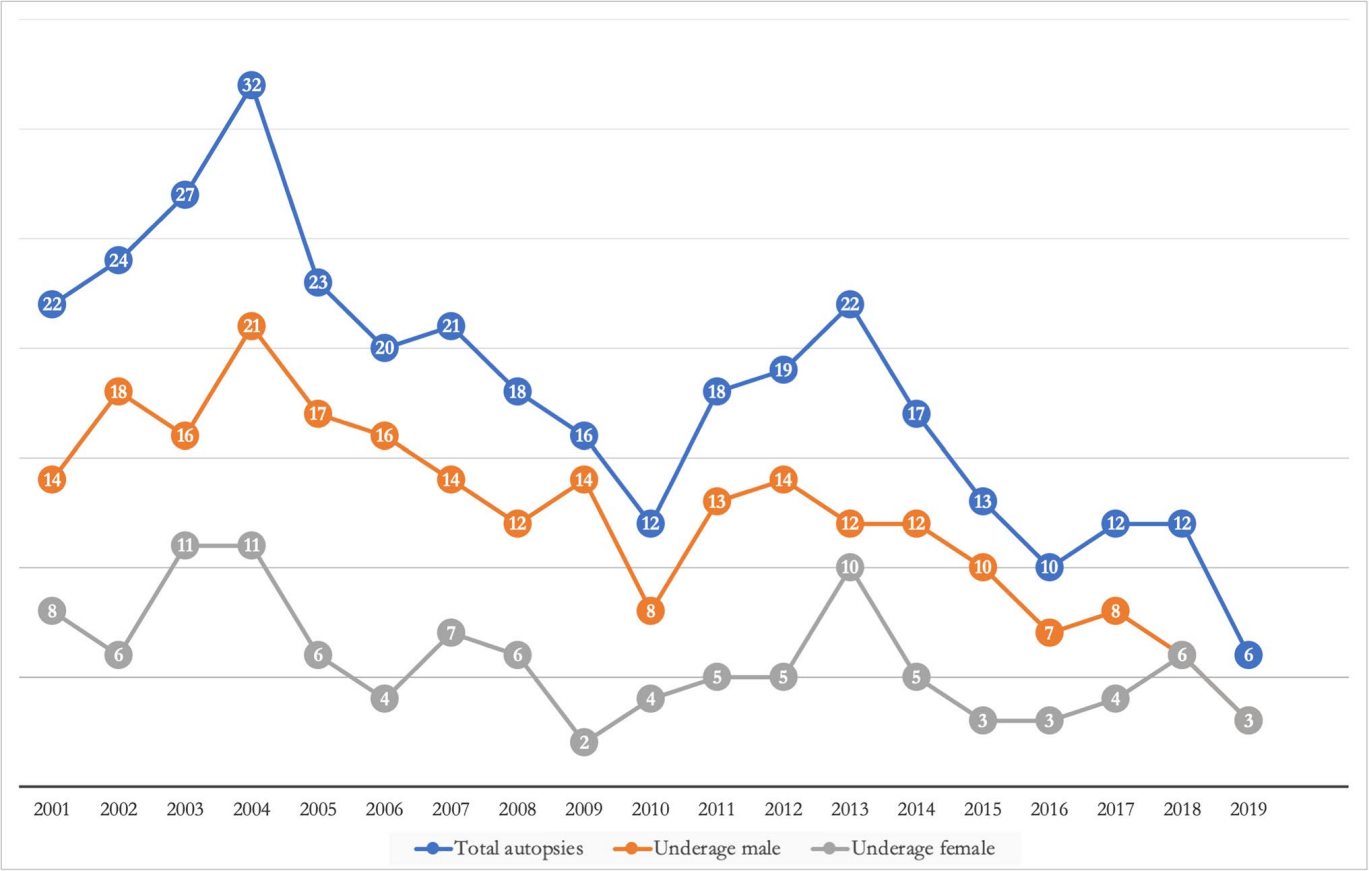
Number of minors autopsied at the Institute of Forensic Medicine in Milan from 2001 to 2019, with the breakdown into males and females for each year

During the 19-year period (2001–2019), 344 autopsies of children and adolescents were performed at the Institute of Forensic Medicine in Milan. This represents 2% of all autopsies in the same period (more than 15.000). On average, only 18.10 ± 6.1 autopsies were performed in minors per year between 2001 and 2019. The highest number of cases was observed in 2004 (32 cases) and the lowest in 2019 (6 cases). Thus, there was a general downward trend of 80% approximately from 2004 to 2019. Overall, the autopsied minor population was composed of 235 males (68.3%) and 109 females (31.7%), for a male-to-female ratio of 2:1. In all years, the number of male juveniles autopsied was higher than the number of female juveniles, with the exception of only two years (2018 and 2019) in which the two sexes were equal (Fig. 1).

Of these autopsies, 325 (94.5%) were ordered by the judicial authority, while the remaining 19 cases (5.5%) were clinical autopsies. Of the total number of minors, 267 (77.6%) were of Italian nationality, 22 (6.4%) were from Europe, 16 were from Africa (4.6%), and 8 were from Asia or South America (2.3% each); in the remaining cases, this information was not available.

**Fig. 2:**
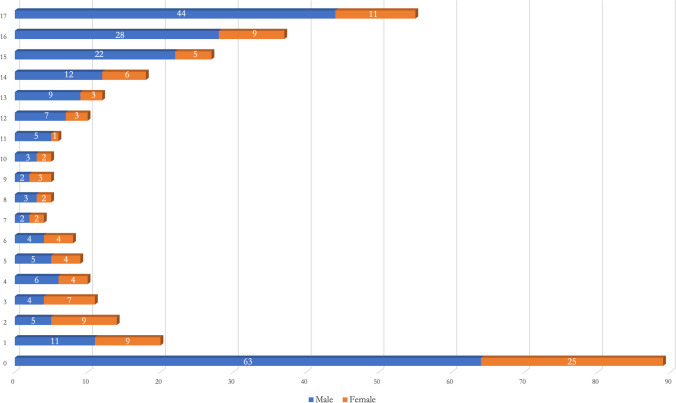
Number of autopsies for each age of deceased minors

The average age of the autopsied minors was 8.9 ± 5.7 years for males and 7.3 ± 6.4 years for females. Considering the number of autopsies performed according to the age of the underage deceased, it was observed that most cases occurred between the ages of zero and 1 year (88 cases, 25.6%), both in males (63 cases, 71.6%) and females (25 cases, 28.4%). Between the ages of 1 and 4 years, 55 cases (16%) occurred, with a slightly higher proportion in females than in males (26, 47%; females, 29, 53%). Between the ages of 5 and 9 years, 31 cases (9%) occurred, with almost equal proportions of male (16 cases, 51.5%) and female (15 cases, 48.5%) victims. Between the ages of 10 and 14, 51 cases (14.8%) were recorded, with most victims being male (36 cases, 70.5%). Between the ages of 15 and 17, the gap widened considerably, with 119 cases (34.6%) registered, of which 94 were males (79%) and 25 were females (21%). Further details are shown in Fig. 2.

The place of death was the home in most cases (123 cases, 35.7%), followed by the street (101 cases, 29.3%) and the hospital (68 cases, 19.7%). In the remaining cases, deaths occurred in public places (22 cases, 6.4%), hotels (10 cases, 3%), bodies of water (8 cases, 2.3%), and the workplace (5 cases, 1.4%); in the remaining cases, this information was not available.

Regarding the manner of death, accident was the most common (155 cases- 45.1%, 100 males and 55 females), followed by natural death (129- 37.5%, 94 males and 35 females), suicide (37- 10.7%, 31 males and 6 females), and finally homicide (23 cases- 6.7%, 10 males and 13 females). In all manners of death, with the exception of homicide, the male sex was numerically more represented.

In terms of age, the number of deaths from natural causes decreased from 68 in babies until 1 year (52.7%) to 27 between 1 and 4 years (21%) and 7 between 5 and 9 years (5.4%), and then increased slightly to 11 between 10 and 14 years (8.5%) and 16 between 15 and 17 years (12.4%). An opposite trend was observed for suicides and accidents. Suicides occurred in the age group between 5 and 9 years with only 1 case (2.7%) and then increased to 11 cases between 10 and 14 years (29.8%) and 25 cases between 15 and 17 years (67.5%). Accidental deaths, on the other hand, were documented in babies until 1 year with 13 cases (8.5%) and then increased with age: 21 cases between 1 and 4 years (13.5%), 19 cases between 5 and 9 years (12.3%), 28 cases between 10 and 14 years (18%), and 74 cases between 15 and 17 years (47.7%). Homicides were represented in all age groups, with 7 cases in babies until 1 year and between 1 and 4 years (30.4% each), 4 cases between 5 and 9 years (17.4%), 1 case between 10 and 14 years (4.4%), and 4 cases between 15 and 17 years (17.4%).

As for non-natural causes of death, the breakdown of victims by sex showed that suicide was a phenomenon that affected the male sex much more frequently than the female one and the first case was observed as early as age 9. In homicides, both sexes were affected, although females were generally more frequently involved, especially up to the age of 4 years and between 15 and 17 years; overall, most homicides were committed between the ages of 0 and 9 years. Finally, deaths from accidents affected all age groups, with the difference between males and females increasing with age (the only exception was the 5–9 age group). Further details are shown in Table 1.

**Table 1:** Manner of death in relation to gender and age group, with details of the raw number and percentage value (%) of the total in each category

	0 y/o		1–4 y/o		5–9 y/o		10–14 y/o		15–17 y/o		Total
	Male	Female	Male	Female	Male	Female	Male	Female	Male	Female	
Natural death	53 (84)	15 (60)	11(42.3)	16(55.2)	5(31.2)	2 (13.3)	11 (30.5)	0 (0)	14 (15)	2 (8)	129
Suicide	0 (0)	0 (0)	0 (0)	0 (0)	1 (6.3)	0 (0)	8 (22.2)	3 (20)	22 (23.4)	3 (12)	37
Homicide	2 (3.3)	5 (20)	4 (15.4)	3 (10.3)	2(12.5)	2 (13.3)	1 (2.8)	0 (0)	1 (1)	3 (12)	23
Accident	8 (12.7)	5 (20)	11(42.3)	10(34.5)	8 (50)	11(73.4)	16 (44.5)	12 (80)	57 (60.6)	17 (68)	155
Total male/female	63 (100)	25 (100)	26 (100)	29 (100)	16 (100)	15 (100)	36 (100)	15 (100)	94 (100)	25 (100)	344
Total per age range	88		55		31		51		119		

**Fig. 3:**
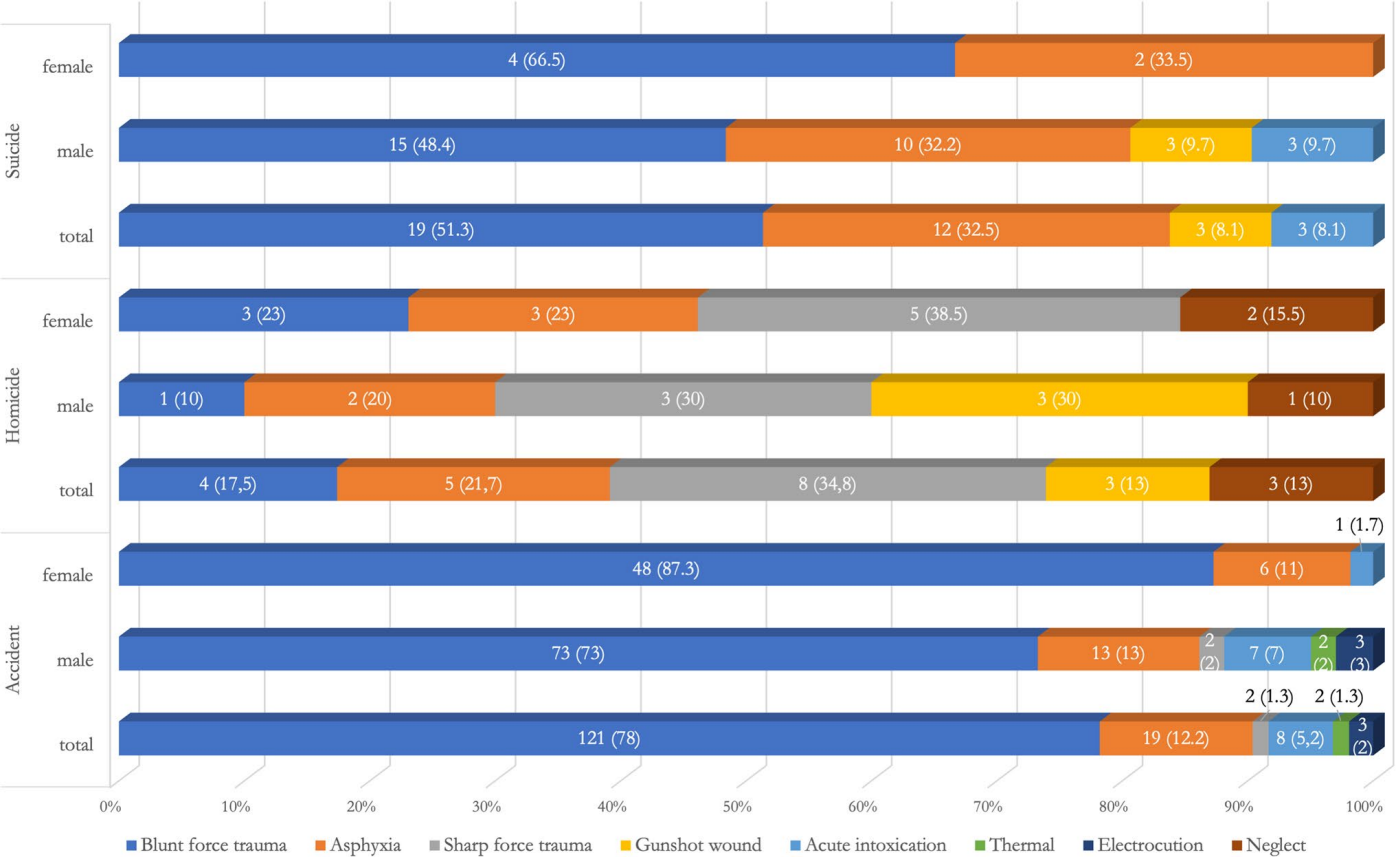
Total methods of injury for suicide, homicide and accidental event, broken down by gender of victims, with details of raw number and percentage value (%) of the total in each category

A detailed examination of all non-natural causes of death (215 cases in total, 141 males and 74 females) revealed that blunt force trauma was by far the most common method of injury (144 cases, 67.0%), followed by asphyxia in 36 cases (16.7%), acute intoxication in 11 cases (5.1%), sharp force trauma in 10 cases (4.6%), firearm in 6 cases (2.8%), electric injury and neglect in 3 cases each (1.4% each), and thermal injury in 2 cases (1%). Breaking down methods of injury by manners of death, it was observed that accidental deaths were most frequently caused by blunt force trauma (traffic accidents – 84 cases, and falls – 37 cases), followed by asphyxia (drowning – 11 cases, and food inhalation – 8 cases), acute intoxication (overdose of illicit drugs – 8 cases), electrical injuries – 3 cases, thermal injuries – 2 cases, and sharp force trauma – 2 cases. Similarly, among suicides, the most common method of injury was blunt force trauma (fall from height – 19 cases), followed by asphyxia (hanging – 7 cases, plastic bag suffocation – 5 cases); other methods consisted of gunshot wounds – 3 cases, and acute intoxication (CO poisoning – 3 cases). In contrast, most homicides were due to sharp force trauma – 8 cases, and asphyxia (choking – 1 case, strangulation – 2 cases, and suffocation – 2 cases), followed by blunt force trauma – 4 cases, gunshot wounds – 3 cases, and neglect – 3 cases (Fig. 3).

Relating the various methods of injury to the age of the victims, it was observed that blunt force trauma always took the first place in every age range. This was particularly striking in victims younger than 4 years and in victims 14 years or older. Asphyxia was the second most common finding across all age groups, affecting both children and adolescents. Acute substance intoxication was recorded in the range of 10 to 17 years; sharp force trauma, electrocution, and thermal injuries predominated between 15 and 17 years. All cases of neglect involved newborns or infants. When the methods of injury were considered by both victims’ age and manner of death, it was apparent that blunt force trauma predominated among accidental deaths in all age groups, followed almost without exception by death due to asphyxia. Ages 1 to 4 years also recorded the first case of accidental electrocution, while ages 10 to 14 years recorded accidental deaths from sharp force trauma and acute intoxication. These injury patterns continued from 15 to 17 years of age, with accidental thermal injuries also occurring. The first case of suicide was a 9-year-old child who died by asphyxia (hanging); between 10 and 14 years, the number of suicide cases increased to 11, all of them carried out through blunt force trauma and asphyxia exclusively. Between the ages of 15 and 17 years (25 cases), a more differentiated framework emerged: in addition to blunt force trauma (which was predominant), suicidal deaths by asphyxia, sharp force trauma and gunshot wounds were also recorded. Among homicides, blunt force trauma, asphyxia, and gunshot wounds were fairly evenly distributed across all age groups, with sharp force trauma slightly predominating. Neglect also occurred in newborns and infants, while firearm use was seen between ages 1 and 4 and between ages 15 and 17 (Table 2). Focusing on blunt force trauma only, which is by far the most significant in terms of numbers, it was observed that most fatal accidents up to the age of 14 were due to traffic accidents involving minors, mainly as victims of hit-and-run accidents or as passengers in cars. After age 15, car accidents continued to predominate, but there was also an increase in the number of motorcycle accidents involving minors, primarily as drivers. Fatal fall accidents were recorded mainly among 5- to 14-year-olds at play (falls from rides, trees, and less frequently from balconies). Blunt injuries in suicides were exclusively from falls from great heights. Among homicides, there were two cases of shaken baby syndrome in children under 4 years of age; the other cases were blunt injuries from blows with the bare hands combined with blunt tools.

**Table 2:** Manner of death (Suicide, Homicide, and Accident) broken down by method of injury and age group, with details of the raw number and percentage value (%) of the total in each category

	0 y/o			1–4 y/o			5–9 y/o		
	Suic	Hom	Acc	Suic	Hom	Acc	Suic	Hom	Acc
Blunt trauma		1 (14.3)	8 (61.5)		1 (14.3)	19 (90.6)		1 (25)	12 (63)
Asphyxia		1 (14.3)	5 (38.5)		2 (28.6)	1 (4.7)	1 (100)	1 (25)	7 (37)
Sharp trauma		2 (28.6)			3 (42.8)			2 (50)	
Gunshot					1 (14.3)				
Intoxication									
Thermal									
Electrocution						1 (4.7)			
Neglect		3 (42.8)							
Total per manner of death	0	7 (35)	13 (65)	0	7 (25)	21 (75)	1 (4)	4 (16)	19 (80)
Total per age range	20			28			24		

	10–14 y/o			15–17 y/o			Total
	Suic	Hom	Acc	Suic	Hom	Acc	
Blunt trauma	6 (54.5)	1 (100)	18 (64.4)	13 (52)		64 (86.5)	144
Asphyxia	5 (45.5)		4 (14.3)	6 (24)	1 (25)	2 (2.7)	36
Sharp trauma			1 (3.5)		1 (25)	1 (1.3)	10
Gunshot				3 (12)	2 (50)		6
Intoxication			4 (14.3)	3 (12)		4 (5.5)	11
Thermal						2 (2.7)	2
Electrocution			1 (3.5)			1 (1.3)	3
Neglect							3
Total per manner of death	11 (27)	1 (3)	28 (70)	25 (24)	4 (4)	74 (74)	215
Total per age range	40			103			

From the ISTAT (National Institute of Statistics) survey it was observed that about 2000 minors (0–17 years) died in Milan (surroundings included) between 2001 and 2019. This number included deaths due to both natural and non-natural causes. The data of the two extreme years were assessed: in 2001, 88 minors died and 22 (25%) were autopsied; in 2019, 71 died and only 6 (8%) were autopsied. Therefore, a decrease of about 70% was recorded.

An in-depth look at deaths due to non-natural causes alone in minors between 2015 and 2019 in the Milan region revealed 86 deaths. A comparison with data from the Institute of Forensic Medicine showed that of these victims, only 33 underwent a forensic autopsy. It followed that the remaining 53 cases (about 61%), although they died of non-natural causes, were not examined by a forensic pathologist. Details on these cases was taken from ISTAT forms, as reported by the physicians who filled them in. In some cases, the information was missing. Specifically, the cases involved 35 children aged 0 to 9 years (66%) and 18 aged 10 to 17 years (34%). Overall, 25 cases (47%) were fatal accidents, 17 cases (32%) were suicides, and 11 cases (21%) had no information. Finally, blunt force trauma was reported in 31 cases (59%), asphyxia in 14 cases (26%), thermal injury in 2 cases (4%), and no information was available in the remaining 6 cases (11%).

## Discussions

The performance of autopsies on minors (children and adolescents) is a practice that may not be performed routinely, or which tends to be discouraged. It is too often a procedure considered “inappropriate” as it maims the innocence of younger victims, according to certain cultures [13]. However, minors are among the most vulnerable population groups, and only through autopsy and associated laboratory analyses it is possible to detect cause of death and even the most deceitful or unintentional or voluntary crimes [14]. In all cases, but especially in the case of underage victims of non-natural death, there is a duty to pursue detailed investigations regarding the specific cause and the manner of death, for which the forensic autopsy is an indispensable tool. Nevertheless, it seems that knowledge and awareness of this crucial necessity are low. In our experience, a forensic autopsy is not always ordered even in cases of non-natural deaths of children and adolescents. Furthermore, even in the forensic literature it has been acknowledged that underage victims have been largely overlooked, especially as regards forensic autopsies. For these reasons, we conducted a study to analyze the case records of autopsies of minors (under 18 years of age in Italy) performed at the Institute of Forensic Medicine in Milan during the period 2001–2019.

A total of 344 autopsies were performed on minors, but a decrease of 80% was observed over the years, from 2004 to 2019. This figure is extremely concerning, as it does not indicate a decrease in the number of minor deaths, but rather a trend toward fewer and fewer autopsies for deaths of children and adolescents. However, it has been shown that when performed, in most cases it was a forensic autopsy (approximately 95%). In addition to the fact that male victims predominated among autopsied minors (68%), interesting data emerged from the age group assessment: approximately 26% of the victims were less than 1 year old, 25% were between 1 and 9 years old, and 49.5% were between 10 and 17 years old. This distribution is consistent with the data in the literature. Indeed, the vast majority of autopsies of children and adolescents are performed on victims who are less than 2 or 3 years old or older than 14 years [13]. This also confirms that the age range between 4 and 9 years is the most often overlooked one, confirming the findings in the literature [13]. Furthermore, not surprisingly, in most cases the home was the place where death from non-natural causes of minors occurred. This finding is fully consistent with the fact that the home is very often the place where direct or indirect violence is perpetrated against minors [2], as is also the case for other vulnerable population groups, such as the elderly [20, 21] or women [22].

Accidents were the most common manner of death (45.1%), followed by natural death (37.5%), suicide (10.7%), and homicide (6.7%). For all manner of death, except homicide, the male sex predominated over the female one. In particular, the greater involvement of the male sex in suicides was also evident from the study by Sauvageau et al., the pioneer in the field of evaluating forensic autopsies in non-natural deaths in minors [13]. The different manners of deaths were then related to age groups. At ages 0 to 1 year and at ages 1 to 4 years, the most common manner of death was natural death, followed by accident and homicide. Overall, accident remained the leading manner of death from age 5 years, with predominance increasing with age, especially in the male sex. In the 14- to 17-year-old age group, suicide took over the second place, reducing natural deaths to the third one. Homicides were recorded primarily in the 0- to 9-year-old age group and consistently ranked last in frequency for all the ages. Compared with the Sauvageau et al. study, we observed fewer homicides overall. This is a very reassuring result, which is also completely consistent with the number of homicides in the general population that are routinely autopsied at our institute [23]. On the other hand, the percentage of underage homicide victims in our study (6.7%) was slightly higher than the one in the study by Terranova et al., in which the authors investigated the phenomenon of homicides against minors in the whole Italian territory, assessing it at 4% approximately [24]. This small difference is quite understandable considering that the study by Terranova et al. only considered the period from 2007 to 2015, while in our study we assessed a wider period from 2001 to 2019, and only the city of Milan. Since this is only one city, albeit a large one, the results obtained cannot and should not be considered representative of the entire national territory.

Regardless of the manner of death, blunt force trauma predominated (67%), followed by asphyxia (about 16.7%), acute intoxication (5.1%), and sharp force trauma (about 4.6%). Most importantly, blunt injuries affected all age groups and always ranked first, especially among children younger than 4 years and older than 14 years. Asphyxia ranked second and affected mostly children, but also adolescents. However, it was very interesting to relate the recorded methods of injury with both the manner of death and the victims’ age. Not only was the general prevalence of blunt force trauma in accidental deaths noted, but also the high number of fatal traffic accidents involving minors, both as passengers and pedestrians at younger ages and as drivers (of cars and motorcycles) at older ages, confirming a phenomenon of worldwide significance [25]. Asphyxia ranked second among all accidental deaths, and other alarming data were uncovered: the first case of accidental electrocution at age 1 to 4 years, the first cases of accidental acute intoxication and sharp force trauma at age 10 to 14 years, and the first case of accidental thermal injuries at age 15 to 17 years. This illustrates that even very young children are exposed to risks that could be avoided through better prevention, the introduction of safety systems and better road traffic awareness. Equally relevant is the fact that the earliest age of suicide was 9 years, and the number of suicides increased to 11 between 10 and 14 years and to 25 between 15 and 17 years. Suicidal acute intoxication and gunshot wounds were recorded in addition to blunt injuries and asphyxia, which were the most common over all the age groups. Undoubtedly, these are also preventable deaths, but it is clear that the risk of suicide should not be underestimated even in very young people [26]. This need is also confirmed by other literature data, in which 6 suicides were recorded in children between the ages of 10 and 14, and 35 suicides in adolescents between the ages of 15 and 19 [13]. In the study by Sauvageau et al., the first and second most common methods of death in suicides were asphyxia and gunshot injuries, while in our study blunt force trauma and asphyxia were mainly observed. Differences were also observed in homicides: in our cases, blunt injuries, asphyxia, and sharp force trauma were fairly evenly distributed across all age groups. Two cases of shaken baby syndrome were recorded, indicating that this vicious form of abuse is still present in today's society despite numerous information and awareness campaigns [27]. In contrast, in Sauvageau et al. study (placed in Canada), homicides with firearms predominated. It is very difficult to interpret these discrepancies, although part of the explanation may lie in the fact that in the study by Sauvageau et al. most of the suicide victims were older, between 15 and 19 years of age [13]. Moreover, in Italy, access to firearms, especially for minors, is extremely difficult. In the light of these findings, we believe that is of great importance to compare case records from various geographical areas, which may highlight distinctions in relation to different cultural and social aspects, and at the same time, also reveal some similarities.

All of these general case data certainly have their own significance, but they are difficult to fully grasp unless they are placed in the geographic context of reference, particularly with respect to the number of minors who die. This type of comparison has never been made in the literature, in part because it requires a detailed knowledge of the epidemiology of the area under study. We were able to perform this type of analysis thanks to our synergy with the ATS, the Local Health Authority, which collects the death records of all deceased in the city of Milan and its surroundings. We were also facilitated by the fact that the Institute of Forensic Medicine is unique in the whole city and the only place where forensic autopsies are performed. Well, a comparison of all minors who died (from any cause) in the Milan area from 2001 to 2019 with the number of minors autopsied showed that there was a 70% decrease (from 22 in 2001 to 6 in 2019). Even more surprising results emerged when, for the years 2015–2019, we examined only the non-natural deaths of minors registered in the same area and compared them with those that underwent a forensic autopsy. It turned out that 61% were not examined by a forensic pathologist. From the data available, they were mainly minors aged 0 to 9 years (66%). Overall, fatal accidents were the most common manner of death (47%), followed by suicides (32%); methods of injury were mostly blunt force trauma and asphyxia. In some cases, this information was missing. Therefore, in all the above-mentioned cases, no forensic autopsy was performed. In other words, our data show not only that fewer and fewer autopsies are performed on minors each year, but also that a relevant percentage of non-natural deaths are not investigated by a forensic pathologist. All of this leads to a dangerous failure to investigate deaths in children and adolescents, which has implications for prevention. It was not possible to compare our data with those of other countries because it is impossible to obtain reliable information on the number of autopsies (especially forensic autopsies and even more in minors) in each country. A single report in the literature, referring to the study by Sauvageau et al. [13], shows that 223 children and adolescents (4–19 years) were autopsied between 2000 and 2004, representing only 6% approximately of all autopsies. Thus, a rather bleak framework of pediatric forensic pathology seems to emerge, as also confirmed by other evidence in the literature, attesting to an often suboptimal manner in which children deaths are being investigated worldwide, such as in Australia [28]. In general, a decreased interest in the assessment of non-natural deaths (across all age groups) has emerged in some European countries, such as the Netherlands, Norway, Denmark, Germany, Austria, and Italy as well [14, 29–31].

Regarding the autopsy of minors in detail, it should be seriously considered that in many countries it may be wrongly rejected due to social, cultural or religious factors, even if the cause of death is not natural. This could apply in whole or in part to Italy as well. Our study shows that 66% of children who died of non-natural causes and were not autopsied were between 0 and 9 years of age, while the percentage dropped to 35% for 10- to 17-year-old age group. So it would seem that the younger the victim, the more likely it is that an autopsy will be overlooked. Therefore, parents/guardians need to be educated about the need for an autopsy in the event of a non-natural death, and forensic examinations need to be strongly considered to better protect vulnerable populations and prevent similar cases in the future. However, communication related to autopsy can be very difficult, especially when parents are in bereavement, and specific limitations to communication include family-specific issues and cultural and socioeconomic barriers. [32]. These issues have not been fully explored, nor have the strategies to overcome them, so it would be beneficial to conduct further studies in this field. However, this type of awareness should also be directed to health and justice professionals so that they know that autopsy is the last bastion to protect the rights of all victims, but especially the most vulnerable age groups (included minors) when prevention and clinic fail [1]. Of course, it is also an extremely important tool for health surveillance and for protecting public health in its broadest sense [14]. With a good understanding of the circumstances, it may be possible to predict and hopefully prevent future deaths in children and adolescents.

Disclosure and targeted seminars, starting with health professionals and the judiciary, could certainly be an important first step in raising awareness of this issue and stopping the current downward trend. In the meantime, since this is not a rapid change, further studies on this issue need to be conducted to fully understand the extent, hopefully in several countries. In addition, further studies are needed in the medium term to assess whether the trend of non-natural deaths in children and adolescents has changed as a result of the recent Covid 19 pandemic, which is known to have had a huge impact on the mental and physical health of billions of people. Preliminary local [23, 33] and cross-national [34] studies do not appear to show an increase in suicides in this context, but this finding needs further investigation.

The study presented is retrospective in nature and therefore has the limitation of being based on available information. However, the lack of information in some of the cause of death report forms is to be understood as a relevant fact underlining the current critical issues, rather than as a limitation of the study.

## Conclusions

This study reveals a new insight into child and adolescent victims. However, in front of the observed decrease in autopsies on minors it is more essential than ever clearly reaffirm that autopsy and any laboratory analysis should be a routine practice in approaching a deceased minor. Whether it is prolonged illness and hospitalization or a sudden and unexpected event due to a natural or exogenous cause, identifying the cause and manner of death is fundamental for the adoption of proper child protection measures. However, our data shows that less and less forensic autopsies are being performed over the last years, which indicates a dangerous lack of investigation of infant and adolescent deaths. This trend must be reversed without further delay and future research perspectives must consist of deepening the study of minors who died of violent causes and did not undergo an autopsy, as they would certainly have been worthy of a medico-legal assessment. Of these, moreover, it would be important to evaluate clinical information and previous visits to emergencies departments in local hospitals. Since there is much that can be learned from the dead to contribute to the welfare of the living, such an evaluation could and should also be applied on frail living subjects, in order to intercept cases of violence and ill-treatment that could result in death.

## Data Availability

All the data have been reported in the manuscript.
